# Climate Change Vulnerability of Native and Alien Freshwater Fishes of California: A Systematic Assessment Approach

**DOI:** 10.1371/journal.pone.0063883

**Published:** 2013-05-22

**Authors:** Peter B. Moyle, Joseph D. Kiernan, Patrick K. Crain, Rebecca M. Quiñones

**Affiliations:** 1 Center for Watershed Sciences and Department of Wildlife Fish and Conservation Biology, University of California Davis, Davis, California, United States of America; 2 Fisheries Ecology Division, Southwest Fisheries Science Center, NOAA National Marine Fisheries Service, Santa Cruz, California, United States of America; 3 ICF International, Sacramento, California, United States of America; 4 Lehrstuhl für Aquatische Systembiologie, Technische Universität München, Freising, Germany; University of Sydney, Australia

## Abstract

Freshwater fishes are highly vulnerable to human-caused climate change. Because quantitative data on status and trends are unavailable for most fish species, a systematic assessment approach that incorporates expert knowledge was developed to determine status and future vulnerability to climate change of freshwater fishes in California, USA. The method uses expert knowledge, supported by literature reviews of status and biology of the fishes, to score ten metrics for both (1) current status of each species (baseline vulnerability to extinction) and (2) likely future impacts of climate change (vulnerability to extinction). Baseline and climate change vulnerability scores were derived for 121 native and 43 alien fish species. The two scores were highly correlated and were concordant among different scorers. Native species had both greater baseline and greater climate change vulnerability than did alien species. Fifty percent of California’s native fish fauna was assessed as having critical or high baseline vulnerability to extinction whereas all alien species were classified as being less or least vulnerable. For vulnerability to climate change, 82% of native species were classified as highly vulnerable, compared with only 19% for aliens. Predicted climate change effects on freshwater environments will dramatically change the fish fauna of California. Most native fishes will suffer population declines and become more restricted in their distributions; some will likely be driven to extinction. Fishes requiring cold water (<22°C) are particularly likely to go extinct. In contrast, most alien fishes will thrive, with some species increasing in abundance and range. However, a few alien species will likewise be negatively affected through loss of aquatic habitats during severe droughts and physiologically stressful conditions present in most waterways during summer. Our method has high utility for predicting vulnerability to climate change of diverse fish species. It should be useful for setting conservation priorities in many different regions.

## Introduction

Freshwater fishes are in decline worldwide because of human-caused degradation of aquatic habitats [1]–[4]. Anthropogenic climate change is further accelerating declines of many freshwater fish species, particularly in regions with arid or Mediterranean climates [5]–[7]. These rapid declines are a major conservation challenge, requiring setting priorities for conservation and for devising strategies to prevent widespread extinctions [8]. One factor hindering development of conservation strategies is limited literature on biology and status of most fishes, especially endemic species of little economic value. Consequently, there is a need for a rapid and repeatable assessment method that can incorporate expert knowledge to determine relative vulnerability of different species to climate change [2], [9]. Here we present a methodology that allows systematic evaluation of climate change impacts on freshwater fishes, using the complete California fish fauna as an example.

Our methodology was developed for California because of our familiarity with the fish fauna, which is reasonably well documented, and because native fishes are known to be in decline [6], [10], [11]. Most (63%) of the 129 native species are endemic to the state, while an additional 19% are shared with just one neighboring state [6], [10], reflecting that zoogeographic and political boundaries largely coincide in California. California has also been invaded successfully by at least 50 alien species of fish [10]. Moyle et al. [6] reported that about 80% of California’s native fishes are either extinct, threatened with extinction in the near future, or vulnerable to extinction if present trends continue. Although about one fish per year has been listed under state and federal endangered species acts for the past 20 years, there are a number of other fishes that likely merit listing [6]. Climate change is clearly exacerbating conditions for native fish species, leading to further declines in California [6], [11]. Although Quiñones and Moyle [12] and similar reviews (e.g., [13]) provide background and discussion on how various climate change scenarios will likely affect fishes and aquatic ecosystems, they do not include predictions for individual species that can be used for conservation planning at various scales, from local to statewide.

Our objectives were to (1) describe a methodological framework for evaluating potential effects of climate change on freshwater fish species, (2) apply the method to assess vulnerability of all native and alien fish species in California, (3) evaluate inter-expert agreement and repeatability of our method, (4) compare each species vulnerability to extinction with and without climate change, and (5) assess taxonomic patterns of vulnerability. Assumptions concerning effects of climate change on California’s aquatic ecosystems are based on models and predictions in Knowles and Cayan [14], Miller et al. [15], and Null et al. [16], and on studies reviewed in Moyle et al. [7] and Quiñones and Moyle [12]. We use the term *vulnerability* to mean likelihood of extinction in the next 100 years. Thus, vulnerability to climate change reflects our assessment of the likelihood that the species will go extinct in the next century, at least partly as the result of climate change effects such as higher water temperatures, extended droughts, or altered stream flow patterns.

## Methods

The methods presented here were modified from the framework developed by Galbraith and Price [17], [29] to assess vulnerability of threatened and endangered vertebrate species (those listed under the United States Endangered Species Act of 1973) to climate change. We adopted their basic methodology because it is repeatable, transparent to users of the information generated, focused on species, and applicable (for sake of comparisons) to more than just fishes. However, we modified their component metrics to make them better suited for evaluating a broad suite of freshwater fishes, not just listed species. Our fish species include species, subspecies, evolutionarily significant units (ESU), and distinct population segments (DPS). All of these categories are considered “species” under the U.S. Endangered Species Act of 1973. See Moyle et al. [10] for definitions.

To assess vulnerability, we used 20 metrics [17], divided into two 10-metric modules that could be scored for each fish species. Module 1 (Data Sheet S1) scored the baseline vulnerability of each species to any major change in their environment. This module was designed to show the degree to which species were declining independent of climate change. Module 2 (Data Sheet S2) then scored various physiological, behavioral, and ecological characteristics of the species that would make it more or less vulnerable to climate change. In each module, individual metrics varied in their maximum point value from 3 to 6, depending on their perceived contribution to vulnerability. Total scores for Module 1 (baseline vulnerability; V_b_) potentially ranged from a 10 (critically vulnerable) to 42 (least vulnerable; Table 1), while scores for Module 2 (climate change vulnerability: V_c_) potentially ranged from 10 (critically vulnerable) to 35 (likely to benefit from climate change; Table 2). The 10 individual metrics in each module were scored using professional judgment and information from species accounts in Moyle [10], Moyle et al. [11], and Moyle et al. [18], with additional references consulted as needed. The three aforementioned works provide access to the literature on each species as well as recent assessments of status and population trends. As a check on consistency and repeatability of the methods, the four author independently scored a group of species from the lower Klamath River (Northern California, USA). We chose the Klamath River Basin because it is largely dominated by native fishes and had recently been intensely studied by Quiñones and Moyle [12].

**Table 1:** Scores and categories used to assess baseline vulnerability of California’s freshwater fish fauna.

Category	Score	Description
Critically vulnerable	<18	Species that are at imminent risk of extinction
Highly vulnerable	18–25	Species that are approaching extinction and are likely to be re-categorized as critically vulnerable if their populations or ranges are diminished further
Less vulnerable	26–33	Widespread species that are declining but have large ranges, so have low risk of extinction
Least vulnerable	34–42	Species that have comparatively large and stable (or increasing) populations or ranges

**Table 2:** Scores and categories used to assess the vulnerability of California’s freshwater fish fauna to climate change.

Category	Score	Description
Critically vulnerable	<17	The species is extremely likely to be driven to extinction by year 2100 without conservation measures
Highly vulnerable	17–22	The species is on the path towards extinction as the result of climate change
Less vulnerable	23–37	The species is likely to decline or become more limited in distribution but extinction unlikely by 2100
Least vulnerable	28–32	The species is likely to be relatively unaffected by climate change, with range and populations remaining stable
Likely to benefit from climate change	>32	The species is likely to increase in range and abundance as the result ofclimate change

In total, baseline and climate change vulnerability scores were developed for 121 native species and 43 alien species (Table S1). Seven extinct or extirpated species were excluded from the analyses, as were flannelmouth suckers (*Catostomus latipinnis*) and Central Valley steelhead trout (*Oncorhynchus mykiss*) because of conflicting information about genetic distinctiveness and current status. In addition, seven alien fishes were excluded due to taxonomic uncertainties (three *Oreochromis* ‘species’), recent eradication (*Esox lucius*), or ambiguous status (three aquarium fishes).

### Baseline Vulnerability Metrics

#### 1. Current population size (last 10 years)

Species with small populations are likely to be more vulnerable to extinction than those with larger populations [3]. Because exact numbers were generally unavailable, likely population size at the time of scoring was estimated as belonging to one of six categories: (1) <100, (2) 100–500, (3) 500–1,000, (4) 1,000–10,000, (5) 10,000–50,000, or (6) >50,000. The overlap in numbers in this metric and others reflects that the estimates are not precise, as might be assumed if ranges end with odd number (e.g., 100–499 rather than 100–500). Population size considers only adult and sub-adult fish that are likely to contribute to the breeding population or potential breeding population. In cases where artificial propagation programs exist, as for many salmonid species, population sizes are based on best estimates of the mean annual number of *naturally* spawning fish in California waters during the previous 10-year period.

#### 2. Long-term population trend

Species with declining populations are likely to be more vulnerable to extinction than those with stable or increasing populations. Assessment of long-term population trends were not restricted to any discrete time period (e.g., last 50 years) in order to capture important causes of decline that occurred more than 50 years ago (e.g., construction of major dams) and to permit inferences to be made from disparate data sources (e.g., commercial fisheries, quantitative escapement estimates, and other historical accounts). The categories are: (1) >80% reduction, (2) >50% reduction, (3) >20% reduction, (4) apparently stable, or (5) increasing.

#### 3. Current population trend (last 10 years)

This metric is based on the 10-year period ending at the time of evaluation. The metric has four categories: (1) rapid decline, (2) slow decline, (3) apparently stable, or (4) increasing. The assumption behind this metric is that the more rapid the current rate of population decline, the more likely a species is to go extinct, with or without climate change. Short-term trends are typically harder to detect than long-term trends because of population variability (e.g., [19]); the rating depended more on professional judgment than for long-term population trend (metric 2, above).

#### 4. Long-term range trend

Species that have suffered range (i.e., extent of distribution) contractions in the past are more likely to be vulnerable to extinction than those with stable or expanding ranges. Past contractions in their range indicate that they are already under stress. The time period for assessment of range trend is left open to permit inclusion of all relevant data sources. Additionally, we consider range fragmentation as part of this metric and assume that fragmentation generally increases extinction risk for freshwater fishes. Thus, a species that occupies much of its historic range but persists only in isolated population fragments would receive a lower score than a species whose range is not fragmented. The categories are: (1) >80% reduction, (2) >50% reduction, (3) >20% reduction, (4) apparently stable, or (5) increasing.

#### 5. Current range trend

The current rate of range change, as discussed in metric 4 (above), is based where possible on empirical data or on best estimates for the previous 10 years. The categories are: (1) rapid decline, (2) slow decline, (3) apparently stable, or (4) increasing. There is typically some uncertainty among these scores because most species are not monitored annually. The scoring often relied on professional judgment of the authors or colleagues familiar with the species. Documentation in Moyle et al. [11], [18] indicates significant range declines in many species over the past 10–20 years.

#### 6. Current vulnerability to stressors other than climate change

Many fish species are either vulnerable to, or affected by, multiple stressors such as water diversion, habitat degradation or harvest. To rate the simultaneous effects of multiple stressors on each species, 13 stressor categories were evaluated in a stressor narrative form for each species (Data Sheet S3). Rationales for the stressor categories are provided in Moyle et al. [6]. For each species, stressors were rated high, intermediate or low according to their likely negative impact on the species. The combined ratings fish species’ current vulnerability to multiple stressors other than climate change were scored as: (1) highly vulnerable, (2) vulnerable, or (3) having low or no vulnerability.

#### 7. Future vulnerability to stressors other than climate change

Some fish species may be more vulnerable than others to natural stressors, such as disease or invasive species. A species’ vulnerability to such events could affect its ability to persist. The future vulnerability of a species to stressors other than climate change has three categories: (1) highly vulnerable, (2) vulnerable, or (3) having low or no vulnerability.

#### 8. Life span and reproductive plasticity

In California, short-lived fish species that must spawn annually may be more vulnerable to demographic failures than long-lived species with multiple life history strategies (Moyle 2002). This metric assumes that longer life span and reproductive plasticity improve the probability of species persistence through stressful periods (e.g., extended drought). Therefore, species are scored as: (1) must spawn annually, typical life span <2 yrs, (2) lifespan 2–5 yrs; single life-history, (3) lifespan 4–10 yrs, usually with multiple life-histories, or (4) long-lived, usually mobile species.

#### 9. Vulnerability to stochastic events

Some species, because of a combination of habitat preferences, small population sizes, and limited ranges, may be more at risk from stochastic events, whether natural or anthropogenic, than others. Stochastic events refer to exceptionally large floods, landslides, contaminant spills and other unpredictable large-scale physical or chemical disruptions that prevent a species from completing its life cycle or that severely alter its habitats. Species are scored as: (1) highly vulnerable, (2) vulnerable, or (3) having low or no vulnerability to major stochastic events.

#### 10. Current dependence on human intervention

Increasingly, the fate of each fish species depends on societal values or policy objectives (either of which may change through time). Thus, species that are heavily dependent on human intervention, management, or specific policies (e.g., hatcheries, water management, artificial barriers) are likely to be more vulnerable to extinction. A high level of human intervention indicates that a species is already imperiled. In addition, human intervention, such as captive breeding, may actually select for traits that reduce survival in the wild [12]. Current dependence on human intervention is scored as:

1. Highly dependent: a captive brood stock program or similar continuous effort is required to prevent extinction, or persistence requires continuous management.
2. Dependent: population persistence requires annual monitoring and intervention when needed (e.g., management of barriers, special flows, removal of alien species, establishment of refuge populations).
3. Somewhat dependent: population persistence requires periodic intervention or habitat improvements (e.g., gravel augmentation or habitat restoration). This category may also include species that indirectly benefit from interventions aimed at other species (e.g., lamprey that benefit from various salmonid management actions).
4. Not dependent: populations require no intervention to persist.

The scores generated for the 10 metrics in Module 1 were combined to produce a baseline vulnerability (V_b_) score for each species. While V_b_ scores fall into a continuum, we divided the scores into four categories following Galbraith and Price [17], [29]: critically vulnerable, highly vulnerable, less vulnerable, and least vulnerable (Table 1).

### Climate Change Vulnerability Metrics

#### 1. Physiological/behavioral tolerance to temperature increase

Stream water temperature is often closely linked to air temperature. Significant warming trends have already been documented in aquatic ecosystems for which long-term temperature data are available [20]. Fish species that require cold water, such as sculpins (Cottidae) and trout (Salmonidae), are most likely to be adversely affected by increased water temperatures, leading to reduced ranges. Conversely, fish species that are physiologically or behaviorally tolerant of increased temperatures and/or lowered dissolved oxygen concentrations (e.g., cyprinids or ictalurids) may increase in abundance or range. Evaluations of thermal tolerances for each species are chiefly based on experimental evidence or robust observational data. When such data are unavailable, rankings are based on inferences from closely related species. A species’ tolerance to increasing water temperatures in the ranges predicted by climate change models is categorized as: (1) very low, (2) low, (3) moderate, or (4) high. Under this convention, a species rated “4” may benefit from future change.

#### 2. Physiological and behavioral tolerance to precipitation change

Climate change models generally predict changes in the amount and timing of precipitation throughout California. Perhaps most importantly, the state is expected to experience advancement in the timing of precipitation events and an increase in the ratio of rain to snow [14], [15]. This will result in more high flow events during winter, increased variability in flows, diminished spring snowmelt pulses, and protracted periods of low (base) flows. Such changes in precipitation and flow regimes will likely alter seasonal availability of spawning and rearing habitat for some native fish species (e.g., Chinook salmon, *Oncorhynchus tshawytscha*) and favor fishes (mostly aliens) that can persist during long periods when stream flows are low and intermittent. The more the natural flow regime is altered, the less native fishes will be favored. Physiological and behavioral tolerance to precipitation change is categorized as: (1) very low, (2) low, (3) moderate, or (4) highly tolerant (suggesting a species is likely to benefit from future change).

#### 3. Vulnerability to change in frequency or degree of extreme weather events

Some fishes face greater risk of extinction or reduction in population or range if climate change results in increased frequency, duration, or magnitude of extreme events such as catastrophic floods and prolonged droughts. Fish species are categorized as likely to be: (1) strongly negatively affected, (2) moderately negatively affected, (3) unaffected, or (4) favorably affected by an increase in extreme events.

#### 4. Dispersive capability

Fish species with high dispersal capabilities should be more likely to adjust to climate change than more sedentary species. In this metric, species are rated according to their ability to disperse from areas being adversely affected by climate change and colonize new areas. Dispersive capability is ranked as low, moderate, or high where:

1. A low rating is assigned to species that are unable to disperse, unlikely to move, or move no more than a few kilometers from their natal area without human assistance. This is usually because they occur in very restricted habitats (e.g., pupfish *Cyprinodon* spp., redband trout *Oncorhynchus mykiss* ssp.).
2. A moderate rating indicates that a species may be able to disperse and colonize new habitats in the same general region in which it is native, assuming natural dispersal corridors remain open (e.g., mountain sucker *Catostomus platyrhynchus*).
3. A high rating refers to highly mobile animals that can disperse long distances to other regions, typically by moving through salt water (e.g., Pacific salmon *Oncorhynchus* spp.).

#### 5. Degree of physical habitat specialization

Fish species have a high degree of habitat specialization (i.e., are not flexible in their choice of habitats) for all or part of their life-cycle, so they may be exceptionally likely to decline if their habitats are strongly altered by effects of climate change. This may be true even if they have high behavioral and physiological tolerances to change. Thus a pupfish that has extreme physiological tolerances may still go extinct if springs it inhabits go dry or become too small. In scoring this variable, species are assigned to one of three habitat specialization categories:

1. Highly specialized: species restricted to a well-defined habitat (e.g., rough sculpin *Cottus asperimmus,* confined to sandy-bottomed spring-fed streams).
2. Moderately specialized: species able to tolerate variability within their typical habitats (most fishes).
3. Generalist: species able to exploit a wide variety of habitats, including artificial habitats such as reservoirs (e.g., Sacramento sucker, *Catostomus occidentalis*).

#### 6. Likely future habitat change because of climate change

In this variable, expert opinion is used to judge likely impact of climate change on spatial extent of a species’ main habitats in the next century, as described in Galbraith and Price [17]. For example, riffle sculpin (*Cottus gulosus*) are largely confined to riffle habitats in cold-water streams at mid-elevations; they have limited mobility, so are likely to suffer considerable loss of available habitat as water temperatures increase and flows decrease during drought. These judgments do not have a high degree of accuracy or precision. Rather, they are intended to be reasonable approximations. Many fishes may depend on two or more habitats during their annual or lifetime cycles. For this variable, fish species were scored according to the largest negative effect. For example, if a species has two critical habitats and the putative effects are estimated to be 20% habitat reduction for one and 80% for the other, the higher percentage determines the score. Likely future habitat change by 2100 because of climate change is categorized as: (1) loss of all or most habitat (>50% reduction), (2) some loss (20–50% reduction), (3) no change, (4) some gain (20–50%), or (5) large gain (>50%).

#### 7. Ability of species to shift at same rate as habitat

The spatial distribution of suitable aquatic habitat for a given species may shift in response to climate change. However, because the distribution of habitats for most native fishes is generally limited by topography, they will not be able to shift appreciably in response to climate change, unless it is in an upstream direction (but see next metric). Alien game fishes typically are immune to this limitation because they will be rapidly moved to additional habitats by anglers or agencies or will be able to colonize habitats vacated by native fishes. The likelihood of species being able to shift at the same rate as habitats are scored as: (1) highly unlikely, (2) unlikely, or (3) likely. Most fishes score 1 or 2 here, but this metric helps to distinguish fishes from more mobile fauna, such as birds, when making broad comparisons.

#### 8. Availability of habitat within new range

If fishes could move readily, like many birds, they might find suitable habitats farther north or upstream above barriers. Given the discrete nature of watersheds, it is likely that the only place suitable habitats can be created for native fishes is upstream of present habitats, above barriers, within the same drainage network. However, many upstream habitats may be unsuitable because of their small size, low flows and/or high gradients typical of lower order streams. Where suitable physical habitats exist, they are likely to be already occupied by other native fishes or to be above barriers. Thus, the potential for large amounts of new habitat to be available is slim, even among anadromous species. Availability of habitat within new range (if any) is categorized as: (1) none, (2) limited in extent, or (3) large in extent. Most native fishes score “1” in this metric. Alien fishes can score higher if humans are likely to relocate them to new, artificial habitats (e.g., reservoirs).

#### 9. Dependence on exogenous factors

This variable describes a fish species’ dependence on natural exogenous factors during its life cycle, especially those likely to be affected by climate change. These special exogenous factors can be related to water quality (e.g., narrow temperature range needed for egg incubation), hydrology (e.g., timing and extent of stream flows to trigger spawning or migration), or biology (e.g., availability of specialized prey at key periods). Fish species are characterized as: (1) highly dependent, (2) moderately dependent, or (3) somewhat dependent. Many native fishes (e.g., Clear Lake hitch, *Lavinia exilicauda chi*) score 1 or 2 here because their reproduction is strongly keyed to spring hydrographs typical of regions with Mediterranean climate.

#### 10. Vulnerability to alien species

All kinds of alien species (microbes, plants, invertebrates, and vertebrates) may exacerbate effects of climate change by stressing native fishes through predation, competition, disease, and habitat modification, especially if changing conditions favor the alien species. Carlisle et al. [21] found that throughout the United States, fishes adapted to lake or pond environments tend to dominate fish assemblages of altered streams because of reduced flows, a likely major impact of climate change on California fishes. In California, most alien fishes are adapted to lentic or slow-moving riverine environments [10]. Virtually all aquatic ecosystems in the state host alien species and these species now dominate many habitats and watersheds. Here we characterize inland fish species as being: (1) highly vulnerable, (2) moderately vulnerable, or (3) somewhat vulnerable to known alien species that have invaded or can invade their habitats. In some cases (e.g., Colorado cutthroat trout *Oncorhynchus clarki pleuriticus*) an established but rare alien species may be vulnerable to the invasion of other species.

Each of the above 10 variables are assigned best estimate numerical scores which are summed to produce an evaluation of the species’ potential vulnerability to climate change (V_c_). Following Galbraith and Price [17], [29], we divided scores into five categories: critically vulnerable, highly vulnerable, less vulnerable, least vulnerable, and likely to benefit from climate change (Table 2).

### Evaluating Certainty

Two methods for evaluating certainty/uncertainty were incorporated into the scoring system. First, each metric was assigned a *best-estimate* score and an *alternate* score. The best estimate was derived from empirical evidence or professional judgment of the most likely case. Alternate scores represent less likely, but not unreasonable, estimates for a given metric. Where a best estimate had high certainty, an alternate score was not assigned. Contrasting the total best- estimate score in each module (i.e., sum of the best estimate for all 10 metrics) with highest and lowest score totals (derived from a combination best and alternate estimates) provided an indication of the range of likely status scores likely for the species under consideration.

Second, each best-estimate score was assigned a numeric certainty (confidence) evaluation of high (score = 3), medium (score = 2), or low (score = 1). These ordinal rankings were based on expert judgment concerning the quantity and quality of the information that was the basis of the best estimate scores. Generally, a certainty score of 3 meant the judgment was strongly supported by published reports, especially in the peer-reviewed literature. A score of 2 indicated less information in the primary and secondary literature but strong support in the professional judgment of the authors or other scientists most familiar with the species. A score of 1 indicated limited support in the literature; scoring relied more heavily on professional judgment than for most other species. Total certainty scores (10 metrics) for each module could range from 10 (highly uncertain) to 30 (highly certain). A similar scoring system is presented in Gardali et al. [9].

As an additional check on the accuracy and utility of the assessment methods, we compared results generated for both baseline and climate change vulnerability with status scores Moyle et al. [6] recently developed for native fishes.

### Ethics

Because this paper uses only data developed from other studies, there are no ethical conflicts with animal use.

#### Data availability

All scoring data used in this project is available at http://pisces.ucdavis.edu/resources/climate-change-effects-california-fishes.

## Results

### Vulnerability of California’s Freshwater Fishes

**Figure 1:**
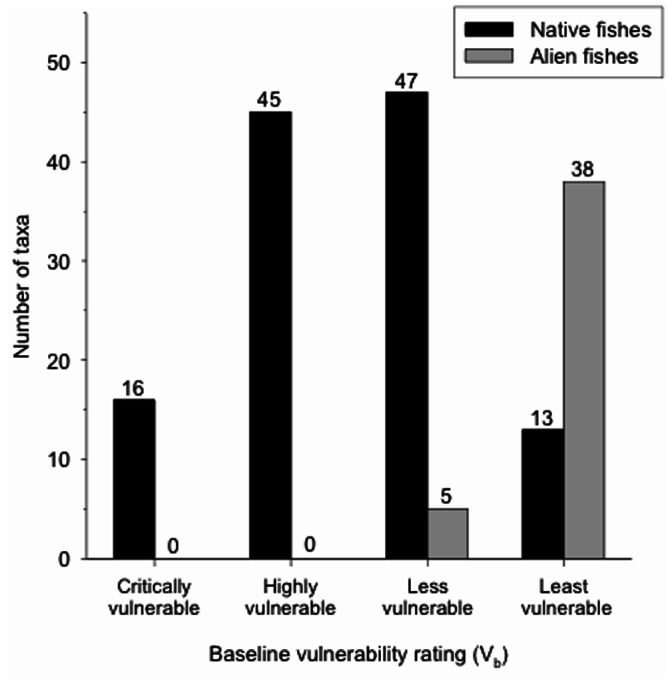
The native and alien fish species in California can be classified into four categories of baseline vulnerability to extinction by 2100. See text for explanation of scoring system and categories.

For baseline vulnerability (V_b_), native fishes had a mean score of 25.7 (±1 standard deviation, SD = 6.1; *N* = 121) and scores for individual species ranged from a low of 13 (Southern Oregon Northern California Coast coho salmon *Oncorhynchus kisutch*) to a high of 37 (Lahontan redside *Richardsonius egregius*). Sixteen native species scored as critically vulnerable, 45 as highly vulnerable, 47 as less vulnerable, and 13 as least vulnerable (Figure 1). In all, nearly 50% of California’s native fish species were categorized as having critical or high vulnerability to extinction, even without considering future climate change. Conversely, alien fishes (*N* = 43) had a mean baseline score of 36.7±3.1, with individual species ranging from 28 (striped bass *Morone saxatilis*, American shad *Alosa sapidissima*, and Colorado cutthroat trout) to 40 (goldfish *Carassius auratus*, channel catfish *Ictalurus punctatus*, and yellowfin goby *Acanthogobius flavimanus*). All but five alien species were categorized as least vulnerable and none showed critical or high baseline vulnerability (Figure 1).

**Figure 2:**
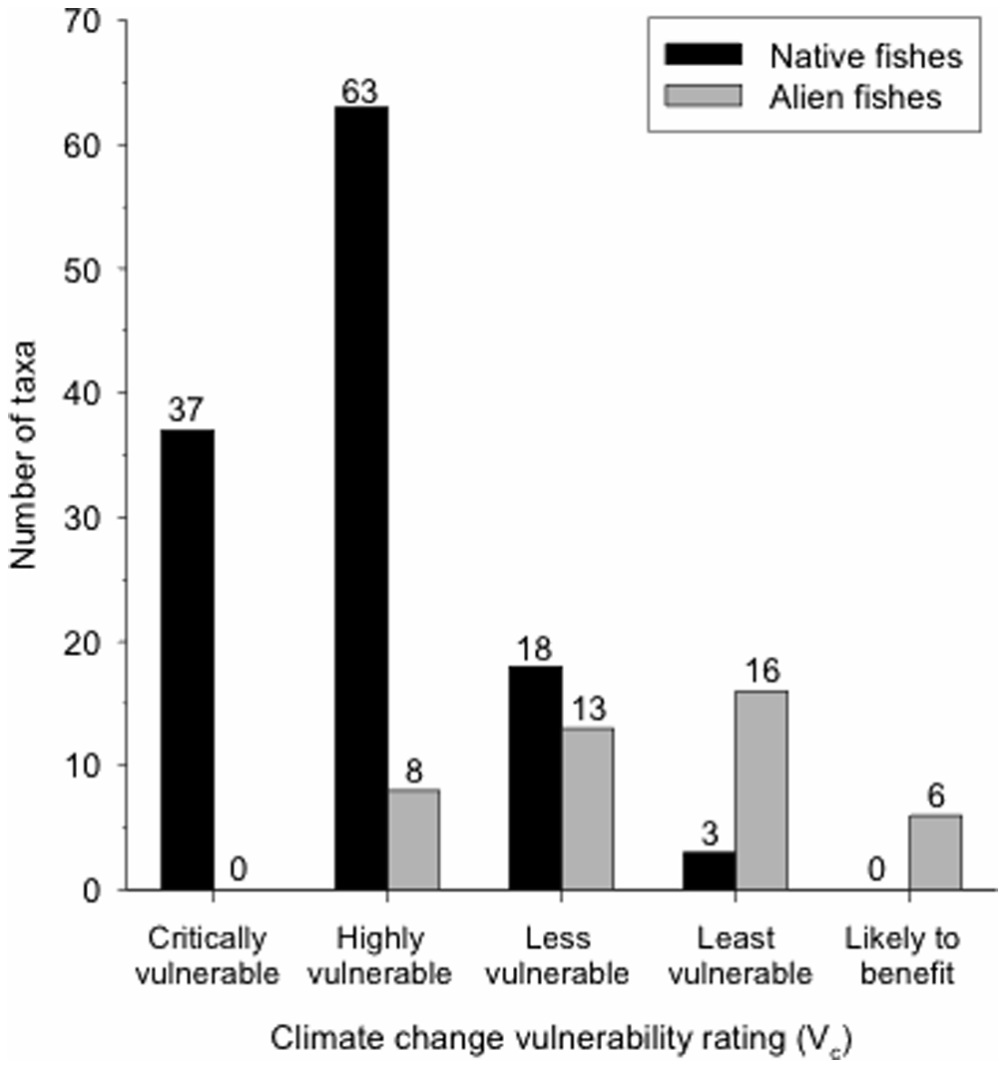
The native and alien fish species in California can be classified into four categories of vulnerability to extinction as the result of climate change by 2100. See text for explanation of scoring system and categories.

For climate change vulnerability (V_c_), native fishes had a mean score of 18.7±4.1. Klamath Mountains Province summer steelhead trout (*O. mykiss*) was the most vulnerable native taxon (V_c_ = 11), whereas staghorn sculpin (*Leptocottus armatus*), a largely estuarine species, was the least vulnerable (V_c_ = 31). Thirty-seven native species scored as critically vulnerable, 63 as highly vulnerable, 18 as less vulnerable, and 3 as least vulnerable (Figure 2). No native species was scored as likely to benefit from climate change. In short, 83% of native fishes were found to be critically or highly vulnerable to climate change. Conversely, alien fishes had a mean V_c_ score of 27.5±4.9 with a range of 17 to 35, for kokanee (*Oncorhynchus nerka*) and black bullhead (*Ameiurus melus*), respectively. None scored as critically vulnerable, 8 as highly vulnerable, 13 as less vulnerable, 16 as least vulnerable and 6 as likely to benefit. Overall, only 19% of alien fishes showed a high vulnerability to climate change, although 86% (37 species) showed at least some vulnerability to climate change, reflecting that aquatic habitat quality and quantity are likely to decline under most scenarios.

### Evaluating Certainty

**Figure 3:**
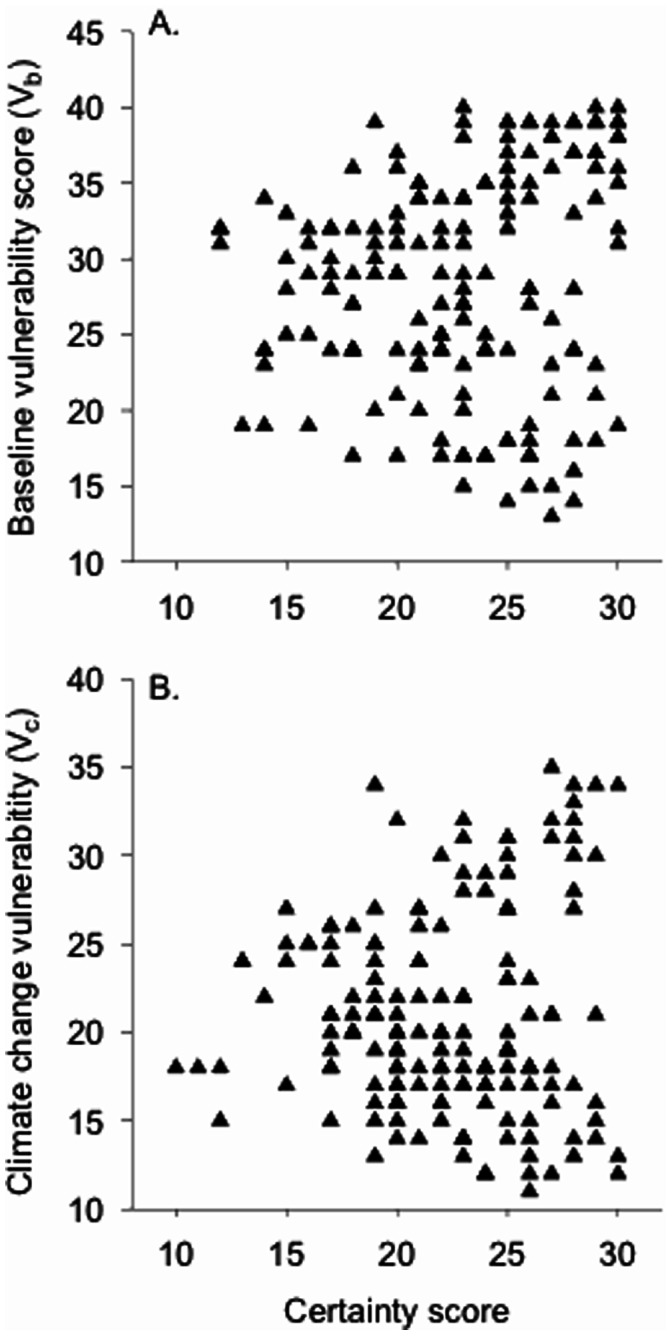
The relationship between certainty scores and baseline and climate change vulnerability scores is weak. Spearman rank correlation for baseline vulnerability (A) is 0.195 (*P* = 0.01) and for climate change vulnerability (B) is −0.041 (*P*>0.05).

Mean certainty scores for the V_b_ and V_c_ modules were 22.8±4.6 and 22.2±4.3, respectively (*N* = 164). These values suggest that scores were assigned to most species with reasonable confidence because the maximum value was 30, the minimum 10 for each module. The closer to 10 that a species is scored, the lower the confidence in the status evaluation of the species. We found a weak but significant positive correlation (non-parametric Spearman rank) between a species’ baseline vulnerability score and the level of certainty associated with that score (*r_s_*_(162) = _0.195, *P* = 0.01; Figure 3A). However, there was no relationship between a species’ climate change vulnerability score and the perceived quality of the information used to generate that score (*r_s_*_(162)_ = −0.041, *P* = 0.60; Figure 3B).

### Inter-expert Agreement and Repeatability

As a test of the consistency and repeatability of our scoring system, the four authors of this study independently completed the two vulnerability modules for 20 species found in the lower Klamath Basin (below Iron Gate Dam) in Northern California, using the same sources of information (e.g., [10]–[12], [18]). Two of the scorers (P.B.M and R.M.Q) had considerable familiarity with the fishes of the basin, while the two others (J.D.K and P.K.C) had more expertise on fishes of other regions. Scores generated independently for both the baseline (Total V_b_; Table 3) and climate change (Total V_c_; Table 4) modules were largely concordant and inter-expert scores ranged from 0 to 7 points, with one exception (i.e., the poorly studied Klamath River lamprey *Entosphenus similis*; Table 3). Nonetheless, given that scores fall on a continuum of vulnerability, it was not uncommon for one or more reviewer to produce a score that resulted in a species being assigned to a different (i.e., higher or lower) vulnerability category.

**Table 3:** Baseline vulnerability (V_b_) scores generated by four independent expert reviewers for native fishes of the lower Klamath River, California.

Taxon	Total V_b_	V_b_ high	V_b_ low	Certainty score	V_b_ rating
Pacific lamprey	21.8 (20–24)	28.5 (27–30)	19.5 (18–21)	20.0 (19–21)	V_b_2
Klamath River lamprey	28.5 (19–32)	31.5 (24–34)	24.8 (15–30)	12.5 (12–13)	V_b_3−
Western brook lamprey	29.3 (26–33)	32.3 (29–35)	24.0 (20–27)	12.3 (10–15)	V_b_3
Northern green sturgeon	27.5 (26–29)	31.8 (29–33)	24.3 (20–27)	19.5 (18–21)	V_b_3
Klamath speckled dace	34.0 (31–35)	35.8 (35–36)	33.3 (31–34)	19.3 (13–24)	V_b_4−
Klamath smallscale sucker	34.0 (33–35)	36.8 (35–39)	32.0 (30–34)	20.5 (17–22)	V_b_4−
Eulachon	17.5 (16–18)	24.0 (23–25)	17.3 (15–18)	21.3 (19–23)	V_b_2−
Upper Klamath-Trinity fall Chinook salmon	22.5 (20–24)	26.5 (23–28)	19.8 (17–21)	25.3 (22–28)	V_b_2
Upper Klamath-Trinity spring Chinook salmon	16.5 (15–18)	19.0 (17–22)	15.3 (13–17)	27.0 (26–28)	V_b_1+
Southern Oregon Northern California coast fall Chinook salmon	24.5 (21–27)	29.3 (26–32)	21.3 (18–24)	22.3 (21–24)	V_b_2+
Southern Oregon Northern California coast coho salmon	13.5 (13–14)	17.5 (16–20)	12.0	26.0 (24–27)	V_b_1
Pink salmon	17.0 (15–19)	24.3 (23–26)	15.5 (14–17)	18.0 (16–19)	V_b_1+
Chum salmon	19.5 (18–22)	25.5 (23–28)	16.8 (16–18)	14.8 (13–17)	V_b_2
Klamath Mountains Province winter steelhead	24.8 (21–27)	28.3 (25–31)	20.5 (18–22)	21.8 (20–23)	V_b_2+
Klamath Mountains Province summer steelhead	16.5 (15–17)	21.8 (21–23)	14.8 (13–16)	23.5 (21–26)	V_b_1
Coastal cutthroat trout	27.3 (25–29)	31.5 (21.32)	23.5 (22–26)	19.0 (17–21)	V_b_3−
Lower Klamath marbled sculpin	32.0	33.0 (32–34)	28.8 (26–31)	14.3 (10–16)	V_b_3
Coastal prickly sculpin	35.5 (33–37)	35.5 (33–37)	29.8 (24–32)	24.5 (21–30)	V_b_4−
Coastrange sculpin	31.8 (31–32)	35.0 (34–36)	27.8 (26–30)	18.5 (16–21)	V_b_3
Coastal threespine stickleback	33.0 (31–35)	35.0 (34–36)	28.5 (27–30)	20.8 (17–23)	V_b_3+

*Notes*: Data are presented as mean scores with ranges in parentheses. Mean total V_b_ scores translate into vulnerability categories as outlined in Table 1: V_b_1 = critically vulnerable, V_b_2 = highly vulnerable, V_b_3 = less vulnerable, V_b_4 = least vulnerable. A plus (+) or minus (−) sign following a V_b_ rating indicates that the total score generated by one or more reviewer resulted in the taxon being assigned to a higher (less vulnerable) or lower (more vulnerable) category, respectively.

**Table 4:** Climate change vulnerability (V_c_) scores generated by four independent expert reviewers for native fishes of the lower Klamath River, California.

Taxon	Total V_c_	V_c_ high	V_c_ low	Certainty score	V_c_ rating
Pacific lamprey	18.8 (17–22)	24.5 (24–26)	14.5 (14–16)	20.8 (19–22)	V_c_2
Klamath River lamprey	17.8 (15–20)	21.5 (21–22)	14.5 (12–17)	12.0 (10–14)	V_c_2−
Western brook lamprey	16.8 (15–18)	22.0 (21–23)	14.0 (13–15)	16.5 (12–19)	V_c_1+
Northern green sturgeon	17.8 (16–20)	21.5 (20–24)	14.3 (13–15)	22.3 (19–24)	V_c_2−
Klamath speckled dace	24.0 (23–25)	29.5 (27–31)	22.5 (21–23)	17.3 (14–20)	V_c_3
Klamath smallscale sucker	26.8 (24–28)	30.8 (30–32)	14.3 (13–15)	22.3 (19–24)	V_c_3+
Eulachon	18.8 (15–20)	24.3 (21–26)	16.0 (11–18)	20.8 (18–24)	V_c_2−
Upper Klamath-Trinity fall Chinook salmon	17.3 (16–18)	21.8 (21–23)	14.3 (13–15)	24.5 (21–27)	V_c_2−
Upper Klamath-Trinity spring Chinook salmon	14.8 (14–15)	19.3 (17–22)	13.8 (13–14)	25.5 (23–29)	V_c_1
Southern Oregon Northern California coast fall Chinook salmon	17.5 (17–18)	20.5 (19–22)	14.3 (13–16)	24.8 (23–26)	V_c_2
Southern Oregon Northern California coast coho salmon	15.0 (14–16)	18.3 (16–21)	13.8 (13–14)	27.3 (24–29)	V_c_1
Pink salmon	17.3 (16–19)	21.5 (19–24)	14.8 (14–15)	20.5 (18–24)	V_c_2−
Chum salmon	17.5 (17–18)	20.5 (19–23)	14.5 (13–15)	21.0 (17–24)	V_c_2
Klamath Mountains Province winter steelhead	20.8 (18–24)	22.3 (18–25)	16.5 (15–19)	25.3 (23–27)	V_c_2+
Klamath Mountains Province summer steelhead	13.0 (11–16)	17.0 (14–21)	11.5 (11–12)	24.5 (23–26)	V_c_1
Coastal cutthroat trout	16.8 (16–18)	22.5 (20–24)	14.0 (13–15)	22.8 (20–24)	V_c_1+
Lower Klamath marbled sculpin	20.3 (19–21)	24.8 (23–26)	17.0 (16–18)	16.3 (10–22)	V_c_2
Coastal prickly sculpin	26.5 (26–28)	28.5 (27–30)	22.3 (18–25)	22.5 (16–28)	V_c_3+
Coastrange sculpin	21.5 (20–23)	24.3 (22–26)	17.0 (12–20)	22.0 (20–24)	V_c_2+
Coastal threespine stickleback	23.8 (22–26)	27.5 (26–29)	20.8 (18–22)	19.0 (15–25)	V_c_3−

*Notes*: Data are presented as mean scores with ranges in parentheses. Mean total V_c_ scores translate into vulnerability categories as outlined in Table 2: V_c_1 = critically vulnerable, V_c_2 = highly vulnerable, V_c_3 = less vulnerable, V_c_4 = least vulnerable. A plus (+) or minus (−) sign following a V_c_ rating indicates that the total score generated by one or more reviewer resulted in the taxon being assigned to a higher (less vulnerable) or lower (more vulnerable) category, respectively. Scientific names of all species can be found in Table S1.

### Baseline vs. Climate Change Vulnerability

**Figure 4:**
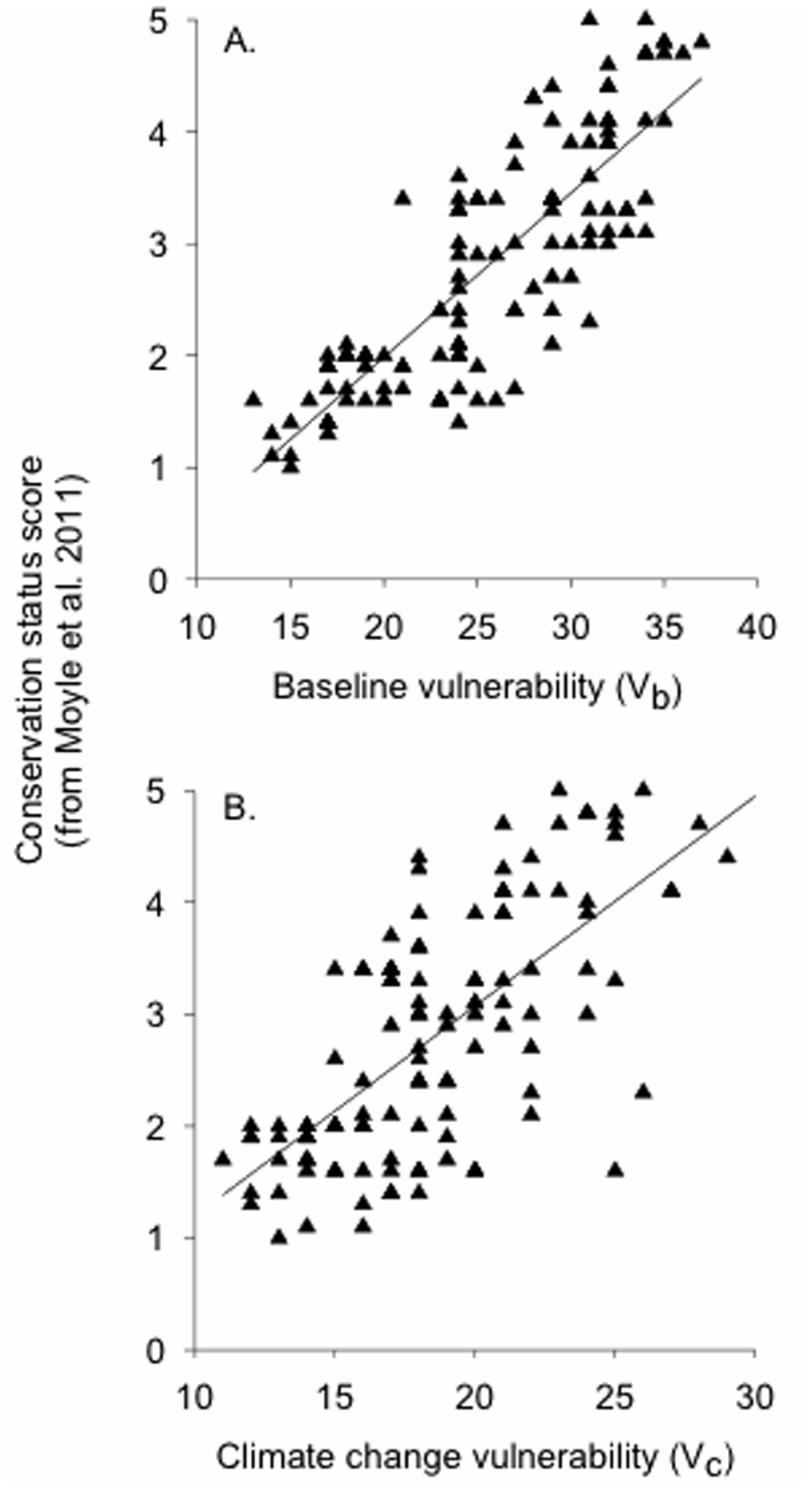
Baseline and climate change vulnerability scores for native California fishes in the present study and conservation status scores reported in Moyle et al. (2011) are highly correlated. In both assessments, lower scores indicate greater vulnerability. Spearman rank correlations are 0.831 and 0.667 for panels A and B, respectively (df* = *118; *P*<0.01 in both cases). *Note*: Moyle et al. (2011) used seven metrics to rate the status of each species of native fish in California on a scale of 1.0 to 5.0, where 1.0 indicated a species on verge of extinction and 5.0 represented a species that was widespread and abundant.

We found a strong positive correlation between the V_b_ and V_c_ scores derived for each species (*r*_s__(162)_ = 0.841, *P*<0.01; data not shown). The V_b_ scores were more strongly correlated with a species’ previously determined conservation status [6] than were the V_c_ scores (V_b_: *r*_s__(118)_ = 0.831, *P*<0.01; V_c_: *r*_s__(118)_ = 0.667, *P*<0.01; Figure 4).

### Taxonomic Comparisons

From a broad taxonomic perspective, fishes in families native to California tended to fare worse in response to climate change than fishes in families not native to California (Tables 5 and 6). In contrast, families dominated by alien species tended to be less affected by climate change. Species in the Centrarchidae, for example, were most likely (92%) to be unaffected by or benefit from climate change. Significantly, the 11 “least vulnerable” centrarchid species are all alien species and the one species rated as highly vulnerable is native (Sacramento perch, *Archoplites interruptus*). Native anadromous species in the families Salmonidae, Acipenseridae, and Petromyzontidae generally showed high vulnerability to climate change and high baseline vulnerabilities. Of the 31 anadromous species, 13 were rated critically vulnerable, 14 as vulnerable, 2 as having low vulnerability and none as being least vulnerable or likely to benefit.

**Table 5:** Baseline vulnerabilities (V_b_) of freshwater fishes found in California by family.

Family	No. taxa	% Native	Baseline Vulnerability Rating (V_b_)							
			Critically vulnerable		Highly vulnerable		Less vulnerable		Least vulnerable	
			*N*	%	*N*	%	*N*	%	*N*	%
Acipenseridae	3	100			2	67	1	33		
Atherinopsidae	1	0							1	100
Catostomidae	14	100	1	7	4	29	7	50	2	14
Centrarchidae	12	8			1	8	1	8	10	83
Cichlidae	2	0							2	100
Clupeidae	2	0					1	50	1	50
Cottidae	12	100			1	8	9	75	2	17
Cyprinidae	45	89	4	9	8	18	18	40	10	22
Cyprinodontidae	8	100	2	25	3	38	2	25		
Embiotocidae	3	100			1	33	1	33	1	33
Fundulidae	2	50					1	50	1	50
Gasterosteidae	4	100	2	50			2	50		
Gobiidae	3	33			1	33			2	67
Ictaluridae	7	0							7	100
Moronidae	2	0					1	50	1	50
Osmeridae	3	100	1	33	2	67				
Percidae	2	0							2	100
Petromyzontidae	7	100			4	57	3	43		
Poeciliidae	3	0					1	33	2	67
Salmonidae	36	86	6	17	17	47	7	19	5	14

**Table 6:** Climate change vulnerabilities (V_c_) of freshwater fishes found in California by family.

Family	No. taxa	% Native	Climate Change Vulnerability Rating (V_c_)									
			Critically vulnerable		Highly vulnerable		Less vulnerable		Least vulnerable		Likely to benefit	
			*N*	%	*N*	%	*N*	%	*N*	%	*N*	%
Acipenseridae	3	100			2	67	1	33				
Atherinopsidae	1	0							1	100		
Catostomidae	14	100	3	21	7	50	3	21	1	7		
Centrarchidae	12	8			1	8	3	25	7	58	1	8
Cichlidae	2	0					1	50			1	50
Clupeidae	2	0			1	50	1	50				
Cottidae	12	100			10	83			2	17		
Cyprinidae	45	89	7	16	15	33	12	27	4	9	2	4
Cyprinodontidae	8	100	2	25	5	63						
Embiotocidae	3	100			3	100						
Fundulidae	2	50			1	50	1	50				
Gasterosteidae	4	100	2	50	1	25	1	25				
Gobiidae	3	33			1	33	1	33	1	33		
Ictaluridae	7	0					3	43	3	43	1	14
Moronidae	2	0			1	50	1	50				
Osmeridae	3	100	2	67	1	33						
Percidae	2	0					2	100				
Petromyzontidae	7	100	2	29	5	71						
Poeciliidae	3	0			1	33			1	33	1	33
Salmonidae	36	86	18	50	17	47						

## Discussion

The method presented here is a tool for systematically predicting the effects of climate change on freshwater fishes. While our metrics were designed specifically to evaluate fishes in a Mediterranean climate region, they should be broadly applicable to fishes (and other aquatic organisms) in other regions. Nevertheless, some modifications of the component metrics may be required, similar to the way we modified metrics of Galbraith and Price [17], [29]. The factor that may most limit application of our method to other regions is apparent lack of information on most species to provide scores for metrics. However, the information-intensive nature of our process does not have to be limiting, if existing published and gray literature is compiled and summarized and if regional experts on fishes are willing to offer informed opinions on the metrics. The metrics’ reliability ratings tell resource which species require further study. As our study shows, differences of opinion on poorly known species (e.g., Klamath River lamprey) can result in different vulnerability scores. For most species, however, different experts arrived at similar scores. Unfortunately, change in aquatic ecosystems is happening so rapidly worldwide [1]–[4] that waiting for high quality information to become available on all species will likely reduce the ability to take action until conservation becomes extremely difficult and expensive.

For California fishes, our estimates of vulnerability to climate change are consistent with the literature on well-studied species in Moyle [10], Moyle et al. [11], and species accounts developed as recent (2013) updates to Moyle et al. [22]. In addition, both baseline and climate change vulnerability scores correlated well with status ratings developed using a different method [6]. This indicates the assessment method can be applied with reasonable accuracy to less studied species, so long as the uncertainty measure of each score is provided.

A potential problem with our method is that the metrics may not be independent of one another, which could bias the total scores upward or downward. Indeed, when correlation (Pearson) matrices were constructed using the 10 variables in each module, 48% of the pairwise comparisons produced correlation coefficients (r) ≥0.5 with 6% exhibiting *r* ≥0.7. This suggests that for a more rigorous analysis of factors affecting trends in the entire fauna, some metrics could be eliminated or new variables developed using a Principal Components Analysis or a similar multivariate approach. However, we chose to retain all 20 variables because we wanted to maximize the amount of information available for assessment of each species; ultimately management decisions will most likely be made at the species level. For the overall analysis, use of variables with some redundancy should help to emphasize differences among species or groups of species, while making causes of status more readily apparent. Aparicio et al. [30] reached a similar conclusion in a study of the biotic integrity of fish assemblages of an Iberian river. Their study compared the value of the two-metric European Fish Index with seven other biotic indices, including a 5-metric index they had developed specifically for the river. They found that the various indices correlated with one another. They also found that indices with more metrics better reflected both causes of trends within the entire system and factors influencing fish diversity at individual sites.

While our metrics were designed to assess species’ status over a large area, they can also at regional scale; species with high vulnerability statewide might have lower or higher vulnerability in some regions and higher vulnerability in others. Regional applications would help managers in specific regions develop strategies to protect the most vulnerable species or groups of species, or even triage species to determine the most effective use of conservation dollars [23].

A comparison of our method with the one that Gardali et al. [9] developed for California birds is instructive because the approaches are similar. They evaluated 358 native bird taxa that earlier studies indicated might be vulnerable to climate change. All were evaluated for their vulnerability using seven metrics, and each scored on a 1–3, 1–4, or 1–5 scale, where a high score indicated high vulnerability. The metrics were divided into two categories: “sensitivity” (four metrics similar to our baseline vulnerability metrics) and “exposure” (three metrics similar to our climate change vulnerability metrics). Gardali et al. [9] then multiplied the totals from the two categories to achieve a final score, considering only birds in the highest 25% of the scores to be vulnerable to climate change. Moderate or high vulnerability was determined as likely for 48 species (13% of all species evaluated).

In contrast, the results of our assessment show that 82% of native fish species were in our two highest categories (critically or highly vulnerable to climate change). Only 19% of alien species showed similar vulnerabilities. Native species were also characterized by greater baseline vulnerability than alien species; 50% of natives scored as critically or highly vulnerable versus none for aliens. While alien species are much more likely to benefit from climate change, many of these species will lose habitat as the result of severe droughts and increasingly stressful conditions in most waterways during summer.

Taken together, this study and that of Gardali et al. [9] indicate that a multi-metric scoring system relying on expert judgment can be used in a geographically distinct region to evaluate general and species-specific trends in vulnerability to climate change for a large number of species within a major taxon. Moreover, the two studies indicate that native freshwater fishes are much more vulnerable to climate change than are birds in the same region. This is because fish are not as widely distributed and have more limited dispersal capabilities. In addition, the s effects that climate change will likely be more severe on freshwater environments because they are already severely impaired.

### Conclusions

Predicted climate change effects on freshwater environments in California will dramatically change the fish fauna. Principally, most native fishes will become more restricted in their distributions and many will ultimately be driven to extinction if present trends continue. In contrast, most alien fishes will suffer much less from climate change but still show some decline, as streams and reservoirs dry up under prolonged drought. Some species (e.g., goldfish, *Carassius auratus*), however, are likely to increase in both abundance and range. Obviously, responses will vary among the two groups; a few natives will also thrive in many streams while some aliens will decline considerably. Beyond these broad conclusions, our study has the following findings:

1. Our method was successful at indicating relative vulnerability of different California fish species to extinction in relations to climate change, as well as showing the susceptibility of the entire native fish fauna to climate change The strength of the method is that it is repeatable by other fish biologists, with scores becoming more reliable as new information is acquired.
2. Fishes with low baseline vulnerability scores, usually because of limited distribution or specialized habitat requirements, are also most likely to have low climate change vulnerability scores. Most fishes with low scores are listed by state or federal agencies as endangered, threatened, or special concern species (Table S1). Some of this baseline vulnerability can be attributed to recent climate change effects, such as warmer stream temperatures.
3. No native fishes are likely to benefit from climate change. Some species, such as Sacramento sucker or Lahontan redside scored as having little susceptibility to climate change but nevertheless may experience declines in the future because of (1) overall decreases in stream habitat, (2) their somewhat lower temperature preferences than many alien fishes, and (3) competition and predation from expanding alien fishes. Putah Creek (Yolo-Solano County, California) may be a model for how streams can be affected by climate change; Kiernan et al. [24] show that if flows released from a dam decrease and change in pattern from the natural flow regime, alien fishes will be favored over native fishes (and vice versa). Presumably increases in late summer temperatures will favor alien species in many streams; even today, the warm lower-most reaches of Putah Creek are largely devoid of native fishes in summer [24].
4. All native anadromous fishes were rated as highly or critically vulnerable to climate change. All members of the Salmonidae were similarly rated as were most other native and alien species Most species requiring cold water (<22°C) habitats. Such fishes already are stressed by other anthropogenic changes to their streams [25]. This is consistent with the findings of Wenger et al. [26].
5. Higher order taxonomy (family level) is a reasonably good predictor of climate change vulnerability. Fishes in the families Cyprinodontidae, Embiotocidae, Osmeridae, Petromyzontidae, and Salmonidae, for example, were almost all highly or critically vulnerable. It is worth noting, however, that the family with the most species (Cyprinidae) had 18 species (both native and alien) scoring in the three categories indicating least vulnerability to climate change.
6. Studies of California streams indicate that different species respond in different ways to variability in flow, which is likely to increase with climate change 19,24. As a result, declining trends may be hard to detect without long-term monitoring. Modifying the flow regime indicates that managing flow regimes in regulated streams may be a powerful tool to counter the negative effects of climate change, as demonstrated by the success of reestablishing native fishes in Putah Creek by [27]. In general, establishment of cool-water refuges for native fish is needed, even in urban streams such as those in the San Francisco Bay region [28].
7. Overall, our study strongly suggests that existing knowledge of California fishes is sufficient to reliably determine which species will need special conservation attention and which will not, as climate change proceeds. In particular, it shows that under present trends, the iconic salmon and other native fishes, will decline while alien fishes increasingly dominate the diminished aquatic systems, assuming present trends are allowed to continue. Understanding these patterns basis should aid development of on statewide and regional conservation strategies to reduce vulnerability to climate change, at least for the next 100 years.
8. Our method is transferrable to other states and regions, using both expert knowledge and literature reviews. The certainty scores are crucial because they show the reliability of the species’ ratings and where research is needed to increase certainty.

## Supporting Information

Table S1 Baseline and climate change vulnerability scores for native and alien fishes in California, as determined by methods discussed in this paper.
(DOCX)

Data Sheet S1 Module 1 score sheet for determining baseline vulnerability of California fishes.
(DOCX)

Data Sheet S2 Module 2 score sheet for determining climate change vulnerability of California fishes.
(DOCX)

Data Sheet S3 Current stressors narrative sheet.
(DOCX)
