# Supplementary material for: Climate Change Vulnerability of Native and Alien Freshwater Fishes of California: A Systematic Assessment Approach
Source: PLoS One. 2013 May 22;8(5):e63883. doi: 10.1371/journal.pone.0063883 (PMC3661749; doi:10.1371/journal.pone.0063883)
Supplement: Table S1 — Baseline and climate change vulnerability scores for native and alien fishes in California, as determined by methods discussed in this paper. (DOCX) [file pone.0063883.s001.docx]

Table S1.Baseline and climate change vulnerability scores for native and alien fishes in California, as determined by methods discussed in this paper.

| Taxon | | | Vulnerability scores | | | | Status score^a^ | Conservation status^b^ |
| --- | --- | --- | --- | --- | --- | --- | --- | --- |
|  |  |  | Baseline | | Climate change | |  |  |
|  |  |  | Best | Range | Best | Range |  |  |
| *Native taxa* | Petromyzontidae | Pacific lamprey, *Entosphenus tridentata* | 21 | 19-28 | 17 | 14-24 | 3.4 | NT |
|  |  | Goose Lake lamprey, *Entosphenus sp.* | 24 | 20-28 | 15 | 13-20 | 2.6 | VU |
|  |  | Klamath River lamprey, *E. similis* | 32 | 30-34 | 18 | 15-21 | 3.9 | NT |
|  |  | River lamprey, *Lampetra ayersi* | 24 | 23-30 | 18 | 16-22 | 3.6 | NT |
|  |  | Kern brook lamprey, *L. hubbsi* | 19 | 16-22 | 15 | 12-18 | 2.0 | VU |
|  |  | Western brook lamprey, *L. richardsoni* | 33 | 27-33 | 18 | 15-22 | 3.1 | NT |
|  |  | Pit-Klamath brook lamprey, *L. lethophaga* | 31 | 30-33 | 18 | 13-23 | 3.6 | NT |
|  | Acipenseridae | Northern green sturgeon, *Acipenser medirostris* | 29 | 27-33 | 18 | 15-21 | 2.4 | VU |
|  |  | Southern green sturgeon, *A. medirostris* | 25 | 19-28 | 25 | 18-26 | 1.6 | EN* |
|  |  | White sturgeon, *A. transmontanus* | 24 | 22-29 | 18 | 17-24 | 2.0 | VU |
|  | Cyprinidae | Thicktail chub, *Siphatales crassicauda* | 0 |  | 0 |  | 0.0 | Extinct |
|  |  | Goose Lake tui chub, *S. t. thalassinus* | 29 | 25-32 | 17 | 14-22 | 3.4 | NT |
|  |  | Pit River tui chub, *S. thalassinus subsp.* | 32 | 29-33 | 24 | 19-27 | 4.0 | LC |
|  |  | Cow Head tui chub, *S. t. vaccaceps* | 24 | 23-29 | 16 | 14-19 | 2.1 | VU |
|  |  | Klamath tui chub, *S. b. bicolor* | 32 | 29-33 | 27 | 25-29 | 4.1 | LC |
|  |  | High Rock Springs tui chub, *S. b. subsp.* | 0 |  | 0 |  | 0.0 | Extinct |
|  |  | Lahontan Lake tui chub, *S. b. pectinifer* | 27 | 25-31 | 19 | 18-23 | 2.4 | VU |
|  |  | Lahontan stream tui chub, *S. b. obesus* | 34 | 33-38 | 25 | 23-28 | 4.7 | LC |
|  |  | Eagle Lake tui chub, *S. b. subsp.* | 33 | 30-35 | 18 | 16-20 | 3.3 | NT |
|  |  | Owens tui chub, *S. b. snyderi* | 17 | 16-22 | 17 | 14-19 | 1.4 | EN* |
|  |  | Mojave tui chub, *S. mohavensis* | 17 | 16-19 | 17 | 15-20 | 1.4 | EN* |
|  |  | Bonytail, *Gila elegans* | 0 |  | 0 |  | 0.0 | Extinct |
|  |  | Blue chub, *Gila coerulea* | 34 | 33-37 | 22 | 19-25 | 3.4 | NT |
|  |  | Arroyo chub, *Gila orcutti* | 31 | 31-34 | 26 | 23-27 | 2.3 | VU |
|  |  | Lahontan redside, *Richardsonius egregius* | 37 | 33-37 | 24 | 23-30 | 4.8 | LC |
|  |  | Sacramento hitch, *Lavinia e. exilicauda* | 24 | 21-31 | 25 | 20-29 | 3.3 | NT |
|  |  | Clear Lake hitch, *L. e. chi* | 25 | 22-30 | 14 | 13-19 | 1.9 | EN |
|  |  | Monterey hitch, *L. e. harengeus* | 29 | 25-31 | 20 | 15-21 | 2.7 | VU |
|  |  | Central California roach, *L. s. symmetricus* | 29 | 22-30 | 24 | 20-29 | 3.4 | NT |
|  |  | Red Hills roach, *L. s. subsp.* | 24 | 21-25 | 16 | 15-19 | 2.0 | VU |
|  |  | Russian River roach, *L. s. subsp* | 31 | 28-33 | 22 | 19-25 | 3.0 | NT |
|  |  | Clear Lake roach, *L s. subsp.* | 31 | 27-35 | 20 | 16-22 | 3.1 | NT |
|  |  | Monterey roach, *L. s. subditus* | 31 | 26-34 | 21 | 13-21 | 3.3 | NT |
|  |  | Navarro Roach, *L. s. navarroensis* | 32 | 31-37 | 24 | 18-27 | 3.0 | NT |
|  |  | Tomales Roach, *L. s. subspecies* | 30 | 27-32 | 18 | 15-20 | 3.0 | NT |
|  |  | Gualala roach, *L. parvipinnus* | 29 | 26-31 | 18 | 13-19 | 3.0 | NT |
|  |  | Northern Roach, *L. mitrulus* | 25 | 22-29 | 17 | 12-19 | 2.9 | VU |
|  |  | Sacramento blackfish, *Orthodon microlepidotus* | 29 | 27-35 | 29 | 24-31 | 4.4 | LC |
|  |  | Sacramento splittail, *Pogonichthys macrolepidotus* | 26 | 25-30 | 21 | 17-26 | 2.9 | VU |
|  |  | Clear Lake splittail, *P. ciscoides* | 0 |  | 0 |  | 0.0 | Extinct |
|  |  | Hardhead, *Mylopharodon conocephalus* | 25 | 22-31 | 15 | 13-21 | 3.4 | NT |
|  |  | Sacramento pikeminow, *Ptychocheilus grandis* | 34 | 30-38 | 23 | 20-27 | 4.7 | LC |
|  |  | Colorado pikeminnow, *P. lucius* | 0 |  | 0 |  | 0.0 | Extinct |
|  |  | Sacramento speckled dace, *Rhinichthys osculus subp.* | 29 | 25-32 | 21 | 18-22 | 4.1 | LC |
|  |  | Lahontan speckled dace, *R. o. robustus* | 35 | 34-36 | 25 | 23-29 | 4.8 | LC |
|  |  | Klamath speckled dace, *R. o. klamathensis* | 35 | 34-35 | 24 | 23-30 | 4.8 | LC |
|  |  | Owens speckled dace, *R. o. subsp.* | 17 | 14-20 | 14 | 11-17 | 1.9 | EN |
|  |  | Long Valley speckled dace, *R. o. subsp.* | 15 | 14-20 | 13 | 12-18 | 1.0 | EN |
|  |  | Amargosa Canyon speckled dace, *R. o. nevadensis* | 23 | 20-28 | 15 | 12-19 | 1.6 | EN |
|  |  | Santa Ana speckled dace, *R. o. subsp.* | 20 | 17-25 | 17 | 17-21 | 1.6 | EN |
|  | Catostomidae | Tahoe sucker, *Catostomus tahoensis* | 34 | 34-37 | 26 | 26-28 | 5.0 | LC |
|  |  | Owens sucker, *C. fumeiventris* | 32 | 32-39 | 24 | 23-27 | 3.9 | NT |
|  |  | Mountain sucker, *C. platyrhynchus* | 29 | 26-29 | 20 | 19-24 | 3.3 | NT |
|  |  | Sacramento sucker, *C. occidentalis* | 31 | 30-37 | 23 | 20-26 | 5.0 | LC |
|  |  | Goose Lake sucker, *C. o. lacusanserinus* | 29 | 29-29 | 22 | 22-24 | 2.1 | VU |
|  |  | Monterey sucker, *C. o. mnioltiltus* | 34 | 28-36 | 23 | 17-24 | 4.1 | LC |
|  |  | Humboldt sucker, *C. o. humboldtianus* | 28 | 29-33 | 21 | 20-25 | 4.3 | LC |
|  |  | Modoc sucker, *Catostomus microps* | 23 | 21-23 | 16 | 14-19 | 1.6 | EN* |
|  |  | Klamath smallscale sucker, *C. rimiculus* | 35 | 34-36 | 27 | 24-31 | 4.1 | LC |
|  |  | Klamath largescale sucker, *C. snyderi* | 19 | 16-23 | 15 | 14-22 | 2.0 | VU |
|  |  | Lost River sucker, *C. luxatus* | 24 | 19-27 | 19 | 19-23 | 1.7 | EN* |
|  |  | Santa Ana sucker, *C. santaanae* | 20 | 18-22 | 17 | 17-18 | 1.7 | EN* |
|  |  | Shortnose sucker, *Chasmistes brevirostris* | 26 | 21-26 | 20 | 19-21 | 1.6 | EN |
|  |  | Razorback sucker, *Xyrauchen texanus* | 17 | 17-20 | 14 | 12-18 | 2.0 | VU* |
|  | Osmeridae | Eulachon, *Thaleichthys pacificus* | 18 | 18-25 | 20 | 18-25 | 1.6 | EN* |
|  |  | Longfin smelt, *Spirinchus thaleichthys* | 20 | 20-27 | 15 | 14-23 | 2.0 | VU * |
|  |  | Delta smelt, *Hypomesus pacificus* | 15 | 13-17 | 12 | 11-13 | 1.4 | EN* |
|  | Salmonidae | Mountain whitefish, *Prosopium williamsoni* | 30 | 26-33 | 21 | 17-22 | 3.9 | NT |
|  |  | Bull trout, *Salvelinus confluentus* | 0 |  | 0 |  | 0.0 | Extinct |
|  |  | Upper Klamath-Trinity fall Chinook salmon, *Oncorhynchus tshawytscha* | 24 | 21-28 | 18 | 15-21 | 2.4 | VU |
|  |  | Upper Klamath-Trinity spring Chinook salmon, *O. tshawytscha* | 16 | 13-17 | 14 | 14-17 | 1.6 | EN |
|  |  | Southern Oregon Northern California coast fall Chinook salmon*, O. tshawytscha* | 27 | 24-32 | 17 | 16-21 | 3.7 | NT |
|  |  | California Coast fall Chinook salmon, *O. tshawytscha* | 23 | 19-26 | 18 | 15-20 | 2.4 | VU * |
|  |  | Central Valley winter Chinook salmon, *O. tshawytscha* | 18 | 16-18 | 12 | 10-14 | 2.0 | VU * |
|  |  | Central Valley spring Chinook salmon, *O. tshawytscha* | 19 | 17-22 | 13 | 11-16 | 2.0 | VU * |
|  |  | Central Valley late fall Chinook salmon, *O. tshawytscha* | 21 | 18-24 | 13 | 11-15 | 1.7 | EN |
|  |  | Central Valley fall Chinook salmon, *O. tshawytscha* | 18 | 17-21 | 16 | 12-17 | 2.0 | VU |
|  |  | Central coast coho salmon, *O. kisutch* | 14 | 14-20 | 16 | 13-19 | 1.1 | EN* |
|  |  | Southern Oregon Northern California coast coho salmon, *O. kisutch* | 13 | 12-17 | 15 | 14-16 | 1.6 | EN* |
|  |  | Pink salmon, *O. gorbuscha* | 17 | 16-24 | 16 | 15-21 | 1.3 | EN |
|  |  | Chum salmon, *O. keta* | 19 | 18-28 | 18 | 15-21 | 1.6 | EN |
|  |  | Northern California coast winter steelhead, *O. mykiss* | 24 | 20-27 | 17 | 16-21 | 3.3 | NT* |
|  |  | Northern California coast summer steelhead, *O. mykiss* | 17 | 16-21 | 14 | 10-16 | 1.9 | EN* |
|  |  | Klamath Mountains Province winter steelhead*, O. mykiss* | 27 | 20-27 | 21 | 19-22 | 3.9 | NT |
|  |  | Klamath Mountains Province summer steelhead*, O. mykiss* | 17 | 16-21 | 11 | 11-14 | 1.7 | EN |
|  |  | Central California coast winter steelhead, *O. mykiss* | 24 | 23-28 | 18 | 15-22 | 2.7 | VU * |
|  |  | Central Valley steelhead*, O. mykiss* |  |  |  |  | 2.4 | VU * |
|  |  | South Central California coast steelhead*, O. mykiss* | 23 | 19-27 | 19 | 17-24 | 2.4 | VU * |
|  |  | Southern California steelhead, *O. mykiss* | 18 | 16-22 | 14 | 13-17 | 1.7 | EN* |
|  |  | Coastal rainbow trout, *O. m. irideus* | 35 | 32-36 | 21 | 17-23 | 4.7 | LC |
|  |  | McCloud River redband trout, *O. m. stonei* | 21 | 18-21 | 12 | 12-16 | 1.9 | EN |
|  |  | Goose Lake redband trout, *O. m. subsp.* | 33 | 31-36 | 17 | 15-21 | 3.3 | NT |
|  |  | Eagle Lake rainbow trout, *O. m. aquilarum* | 24 | 23-25 | 13 | 11-16 | 1.4 | EN |
|  |  | Kern River rainbow trout, *O. m. gilberti* | 19 | 17-21 | 13 | 11-17 | 1.9 | EN |
|  |  | California golden trout, *O. m. aguabonita* | 18 | 15-20 | 14 | 14-17 | 2.0 | VU |
|  |  | Little Kern golden trout, *O. m. whitei* | 23 | 22-26 | 15 | 12-17 | 2.0 | VU * |
|  |  | Coastal cutthroat trout, *O. clarki clarki* | 26 | 26-32 | 16 | 15-20 | 3.4 | NT |
|  |  | Paiute cutthroat trout, *O. c. seleneris* | 27 | 27-28 | 14 | 13-17 | 1.7 | EN* |
|  |  | Lahontan cutthroat trout, *O. c. henshawi* | 18 | 16-21 | 17 | 12-19 | 2.1 | VU * |
|  | Fundulidae | California killifish, *Fundulus parvipinnis* | 31 | 26-32 | 22 | 18-24 | 4.1 | LC |
|  | Cyprinodontidae | Desert pupfish, *Cyprinodon macularius* | 21 | 17-22 | 19 | 15-20 | 1.9 | EN* |
|  |  | Owens pupfish, *C. radiosus* | 17 | 16-17 | 18 | 16-19 | 1.4 | EN* |
|  |  | Saratoga Springs pupfish, *C. n. nevadensis* | 24 | 21-26 | 19 | 15-19 | 2.1 | VU |
|  |  | Amargosa River pupfish, *C. n. amargosae* | 24 | 21-28 | 22 | 17-24 | 2.3 | VU |
|  |  | Tecopa pupfish, *C. n. calidae* | 0 |  | 0 |  | 0.0 | Extinct |
|  |  | Shoshone pupfish, *C. n. shoshone* | 15 | 14-17 | 14 | 11-15 | 1.1 | EN |
|  |  | Salt Creek pupfish, *C. s. salinus* | 28 | 26-30 | 18 | 15-19 | 2.6 | VU |
|  |  | Cottonball Marsh pupfish, *C. s. milleri* | 27 | 24-28 | 16 | 15-20 | 2.4 | VU * |
|  | Cottidae | Rough sculpin, *Cottus asperrimus* | 24 | 22-28 | 16 | 11-20 | 3.4 | NT* |
|  |  | Bigeye marbled sculpin, *C. klamathensis macrops* | 30 | 26-33 | 22 | 20-24 | 2.7 | VU |
|  |  | Lower Klamath marbled sculpin, *C.k. polyporus* | 32 | 31-32 | 20 | 18-23 | 3.3 | NT |
|  |  | Upper Klamath marbled sculpin, *C. k. klamathensis* | 24 | 20-28 | 19 | 17-24 | 3.0 | NT |
|  |  | Coastal Prickly sculpin, *C. asper subsp.* | 36 | 31-36 | 28 | 25-30 | 4.7 | LC |
|  |  | Clear Lake prickly sculpin, *C. a. subsp.* | 32 | 29-33 | 21 | 19-21 | 3.1 | NT |
|  |  | Coastrange sculpin, *C. aleuticus* | 32 | 27-35 | 22 | 18-24 | 4.4 | LC |
|  |  | Riffle sculpin, *C. gulosus* | 29 | 25-32 | 17 | 14-21 | 3.4 | NT |
|  |  | Pit sculpin, *C. pitensis* | 28 | 24-32 | 18 | 16-20 | 4.3 | LC |
|  |  | Paiute sculpin, *C. beldingi* | 32 | 28-33 | 18 | 16-22 | 4.4 | LC |
|  |  | Reticulate sculpin, *C. perplexus* | 31 | 29-32 | 20 | 17-25 | 3.9 | NT |
|  |  | Staghorn sculpin, *Leptocottus armatus* | 35 | 33-35 | 31 | 29-32 |  |  |
|  | Gasterosteidae | Coastal threespine stickleback, *Gasterosteus a. aculeatus* | 32 | 29-34 | 25 | 22-28 | 4.6 | LC |
|  |  | Inland threespine stickleback, *G. a. microcephalus* | 32 | 28-34 | 21 | 15-23 | 4.1 | LC |
|  |  | Unarmored threespine stickleback, *G. a. williamsoni* | 17 | 14-20 | 12 | 10-15 | 1.9 | EN* |
|  |  | Shay Creek stickleback, *G. a. subsp.* | 14 | 13-17 | 12 | 10-14 | 1.3 | EN |
|  | Centrarchidae | Sacramento perch, *Archoplites interruptus* | 23 | 23-25 | 18 | 18-18 | 1.6 | EN |
|  | Embiotocidae | Sacramento tule perch, *Hysterocarpus t. traski* | 25 | 21-30 | 17 | 16-20 | 3.4 | NT |
|  |  | Russian River tule perch, *H. t. pomo* | 34 | 30-35 | 20 | 16-21 | 3.1 | NT |
|  |  | Clear Lake tule perch, *H. t. lagunae* | 27 | 25-31 | 20 | 15-21 | 3.0 | NT |
|  | Gobiidae | Tidewater goby, *Eucyclogobius newberryi* | 24 | 21-27 | 19 | 12-21 | 2.9 | VU * |
| Alien taxa | Clupeidae | American shad, *Alosa sapidissima* | 28 | 27-32 | 19 | 16-23 |  | RD |
|  |  | Threadfin shad, *Dorosoma cepedianum* | 37 | 36-39 | 27 | 25-29 |  | WA |
|  | Cyprinidae | Fathead minnow,*Pimephales promalas* | 36 | 33-38 | 31 | 29-34 |  | WA |
|  |  | Golden shiner, *Notemigonus chrysoleucas* | 39 | 37-41 | 33 | 29-34 |  | WA |
|  |  | Red shiner. *Cyprinella lutrensis* | 39 | 36-39 | 32 | 26-33 |  | WA |
|  |  | Goldfish, *Carassius auratus* | 40 | 39-41 | 34 | 34-34 |  | WA |
|  |  | Common carp, *Cyprinus carpio* | 39 | 38-42 | 32 | 30-34 |  | WA |
|  | Ictaluridae | Channel catfish, *Ictalurus punctatus* | 40 | 39-42 | 27 | 25-30 |  | WA |
|  |  | Blue catfish. *I. furcatus* | 39 | 35-41 | 25 | 24-29 |  | RD |
|  |  | White catfish, *Ameiurus catus* | 38 | 38-41 | 31 | 27-31 |  | WA |
|  |  | Brown bullhead, *A. nebulosus* | 37 | 37-40 | 32 | 31-33 |  | WA |
|  |  | Black bullhead, *A. melas* | 39 | 37-40 | 35 | 32-35 |  | WA |
|  |  | Yellow bullhead. *A. natalis* | 36 | 34-38 | 24 | 21-28 |  | RD |
|  |  | Flathead catfish, *Pylodictus olivaris* | 39 | 38-41 | 32 | 27-34 |  | WA |
|  | Salmonidae | Brook trout, *Salvelinus fontinalis* | 34 | 34-38 | 19 | 15-22 |  | WA |
|  |  | Lake trout, *S. namaycush* | 37 | 34-40 | 18 | 15-20 |  | RD |
|  |  | Brown trout, *Salmo trutta* | 34 | 32-38 | 20 | 17-22 |  | WA |
|  |  | Kokanee, *Oncorhynchus nerka* | 39 | 34-39 | 17 | 16-19 |  | WA |
|  |  | Colorado cutthroat trout, *O. clarki pleuriticus* | 28 | 27-29 | 20 | 16-20 |  | RD |
|  | Atherinopsidae | Mississippi silverside. *Menidia audens* | 38 | 36-38 | 31 | 27-33 |  | WA |
|  | Poeciliidae | Western mosquitofish, *Gambusia affinis* | 38 | 36-39 | 34 | 29-35 |  | WA |
|  |  | Sailfin molly, *Molliensia latipinnis* | 37 | 36-40 | 30 | 28-34 |  | WA |
|  |  | Porthole livebearer, *Poecilopsis gracilis* | 32 | 30-34 | 22 | 17-26 |  | RD |
|  | Fundulidae | Rainwater killifish, *Lucania parva* | 24 | 21-27 | 24 | 21-27 |  | RD |
|  | Moronidae | Striped bass, *Morone saxatilis* | 28 | 26-32 | 23 | 20-24 |  | WA |
|  |  | White bass, *M. chrysops* | 37 | 35-38 | 21 | 20-25 |  | RD |
|  | Percidae | Yellow perch, *Perca flavescens* | 39 | 36-39 | 26 | 21-27 |  | WA |
|  |  | Bigscale logperch*, Percina macrolepida* | 36 | 36-40 | 27 | 22-30 |  | WA |
|  | Centrarchidae | Green sunfish, *Lepomis cyanellus* | 39 | 37-41 | 34 | 31-35 |  | WA |
|  |  | Redear sunfish, *L. microlophus* | 39 | 36-41 | 30 | 26-31 |  | WA |
|  |  | Bluegill, *L. macrochirus* | 39 | 36-40 | 30 | 27-32 |  | WA |
|  |  | Pumpkinseed, *L. gibbosus* | 39 | 37-40 | 31 | 31-34 |  | WA |
|  |  | Warmouth, *L. gulosus* | 35 | 31-40 | 29 | 25-30 |  | WA |
|  |  | Black crappie, *Pomoxis nigromaculatus* | 38 | 36-39 | 27 | 25-30 |  | WA |
|  |  | White crappie, *P. annularis* | 36 | 34-39 | 27 | 25-29 |  | WA |
|  |  | Smallmouth bass, *Micropterus dolomieui* | 37 | 36-38 | 29 | 26-30 |  | WA |
|  |  | Largemouth bass, *M. salmoides* | 39 | 36-41 | 30 | 28-33 |  | WA |
|  |  | Spotted bass, *M. punctulatus* | 38 | 36-38 | 28 | 28-31 |  | WA |
|  |  | Redeye bass, *M. coosae* | 33 | 32-36 | 26 | 25-27 |  | WA |
|  | Cichlidae | California tilapia, *Oreochromis sp.* | 38 | 37-40 | 34 | 29-35 |  | WA |
|  |  | Redbelly tilapia, *Tilapia zilli* | 36 | 32-36 | 27 | 25-29 |  | RD |
|  | Gobiidae | Yellowfin goby, *Acanthogobius flavimanus* | 40 | 35-40 | 26 | 23-29 |  | WA |
|  |  | Shimofuri goby, *Tridentiger bifasciatus* | 35 | 31-37 | 28 | 21-29 |  | WA |

^a,b^ The status score and conservation status designations for native species are from Moyle et al. (2011), based on the standards of the International Union for the Conservation of Nature (IUCN). Status scores of 1.0-1.9 indicate the species is endangered (EN), 2.0-2.9 indicate it is vulnerable to becoming endangered (VU), 3.0-3.9 indicate the species is in decline but not in immediate danger of extinction (near-threatened, NT), and 4.0-5.0 indicate the species of least concern (LC). An asterisk (*) indicates species formally listed under state and/or federal endangered species acts. For alien species, WA indicates a species is widespread and abundant in California (equivalent to LC for native species), RD indicates it has a restricted distribution in fresh water, either by habitat or by watershed (Moyle 2002).
