# Supplementary material for: Climate Change Vulnerability of Native and Alien Freshwater Fishes of California: A Systematic Assessment Approach
Source: PLoS One. 2013 May 22;8(5):e63883. doi: 10.1371/journal.pone.0063883 (PMC3661749; doi:10.1371/journal.pone.0063883)
Supplement: Data Sheet S1 — Module 1 score sheet for determining baseline vulnerability of California fishes. (DOCX) [file pone.0063883.s002.docx]

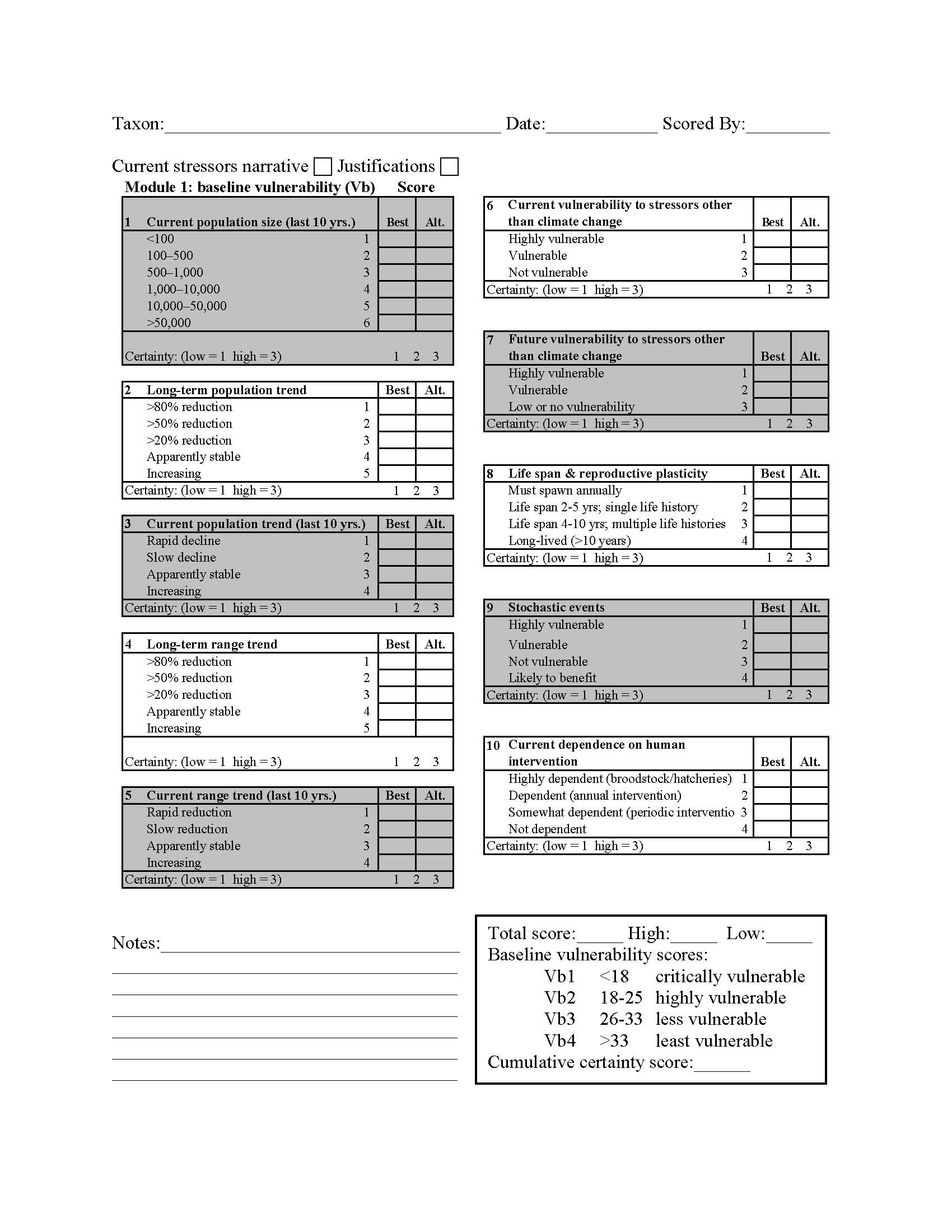


**Data sheet S1.** Module 1 score sheet for determining baseline vulnerability of California fishes. Table S1.Baseline and climate change vulnerability scores for native and alien fishes in California, as determined by methods discussed in this paper.
