# Supplementary material for: Climate Change Vulnerability of Native and Alien Freshwater Fishes of California: A Systematic Assessment Approach
Source: PLoS One. 2013 May 22;8(5):e63883. doi: 10.1371/journal.pone.0063883 (PMC3661749; doi:10.1371/journal.pone.0063883)

**Data sheet S2.** Module 2 score sheet for determining climate change vulnerability of California fishes.
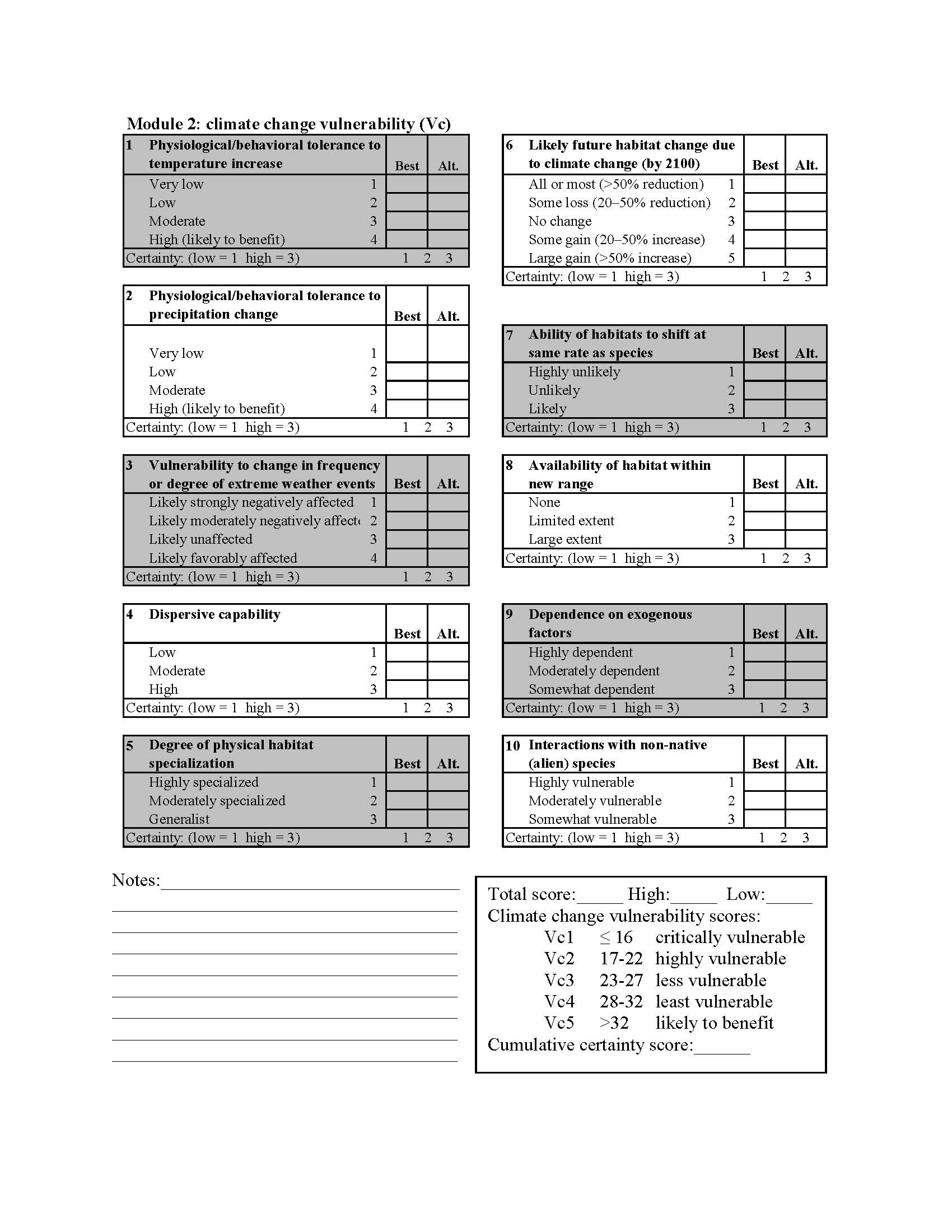

Supplement: Data Sheet S2 — Module 2 score sheet for determining climate change vulnerability of California fishes. (DOCX) [file pone.0063883.s003.docx]
