# Supplementary material for: Climate Change Vulnerability of Native and Alien Freshwater Fishes of California: A Systematic Assessment Approach
Source: PLoS One. 2013 May 22;8(5):e63883. doi: 10.1371/journal.pone.0063883 (PMC3661749; doi:10.1371/journal.pone.0063883)
Supplement: Data Sheet S3 — Current stressors narrative sheet. (DOCX) [file pone.0063883.s004.docx]

**Date sheet S3** Current Stressors Narrative Sheet

Taxon:_________________________________________________Scorer:_____________

On this sheet indicate as *high*, *intermediate*, or *low* the degree to which each stressor currently or potentially limits the viability of fish populations, where a stressor rated “critical” could drive a species to extinction in the near future, a stressor rated “high” is a major limiting factor to population viability, a stressor rated “intermediate” is a factor that has the potential to be a major limiting factor but has had only a moderate effect on population viability to date, and a stressor rated “low” has a low or unknown effect on population viability. “No effect” was used for factors that did not affect the species.

| **Stressor:** | **Rating:** | **Explanation:** |
| --- | --- | --- |
| **Dams & diversions** |  |  |
| **Agriculture** |  |  |
| **Grazing** |  |  |
| **Pollution** |  |  |
| **Urbanization** |  |  |
| **Estuarine alteration** |  |  |
| **Mining** |  |  |
| **Transportation** |  |  |
| **Logging** |  |  |
| **Fire** |  |  |
| **Recreation** |  |  |
| **Harvest** |  |  |
| **Hatcheries** |  |  |
